# Effective Cross-National Respectful Partnerships: A Case Study of Peace Corps’ Volunteer Covid-19 Volunteer Evacuation

**DOI:** 10.5334/aogh.3696

**Published:** 2022-06-30

**Authors:** Jody K. Olsen

**Affiliations:** 1 Former Peace Corps Director, 2018–2021, University of Maryland, Baltimore, US

**Keywords:** global sustainable partnerships, Covid-19 global emergency, health emergency preparedness, international volunteer organizations

## Abstract

Strong, trusted partnerships within a social capital framework are core to Peace Corps’ successful international, national, and community interactions and outcomes. The Peace Corps’ integrated three level model has thrived since its beginning in 1961. During this time, about 250 000 two-year Volunteers have lived and worked in 142 countries. In March 2020, the Peace Corps had to evacuate all 7 000 currently serving Volunteers because of the world-wide Covid-19 pandemic, a task organized and completed in nine days. The evacuation’s success depended on the resiliency of these honored long-term partnerships, and specifically:

- a partnership model with three intertwining layers of collaboration between national and host country staff and Volunteers;
- years of respectful integration of Volunteers in local communities building mutual trust;
- in-country and cross-nation preparedness for health, safety, and security emergencies;
- transparent communication during the evacuation among all involved parties in every nation with Volunteers; and
- in-country and host country staff support across countries during the evacuation.

This case study illustrates elements of effective and sustainable partnerships that ensure their effectiveness during a crisis and survival beyond the crisis.

I write this at the anniversary of one year from my stepping down as the twentieth Director of the Peace Corps. During my tenure, I brought home all 7 000 currently serving Volunteers in nine days as the Covid-19 pandemic spread across countries. This massive – and successful – undertaking was possible because of long-standing and trusted partnerships between the Peace Corps and the communities and countries hosting Peace Corps Volunteers. This is my personal reflection and a reminder to build, honor, and tend relationships with international partners who are collaborators in learning, service, and research in good times and our friends, protectors, and allies in times of crisis.

## Sustainable Partnerships

Sustainable partnerships between 142 countries and the United States over the 60-year history of the Peace Corps have created relationships resilient enough to withstand the total (temporary) withdrawal of all serving Volunteers that happened in March 2020. These partnerships supported the Volunteers returning to the United States, and are now facilitating host country staffs’ continued in-country activities and will support the Volunteers’ safe return to service beginning in 2022.

Sustainable international partnerships are well-defined and include elements of reciprocal relationships and trust. For example, Davy says:

The characteristics that are common to partnerships include voluntary engagement, mutually agreed objectives, distinct accountabilities and reciprocal obligations, and ‘added value’ to what each partner could achieve alone [1].

Another definition suggests five key components of partnership implementation built and maintained over time:

… (i) shared goals, (ii) relations with partners, (iii) capacity for partnership work, (iv) governance and leadership, and (v) trust and trustworthiness [2].

In addition, commitment and conflict resolution are also important characteristics. Mohr and Spekman note:

… [p]artnership characteristics include … attributes of commitment, coordination, and trust; communication quality and participation; and the conflict resolution technique of joint problem solving [3].

Trust, in the case of successful international partnerships, is developed through intentional practices.

Trust accrues from behaving in a trustworthy manner toward others but also requires consistently exuding trust in others. … ensuring that others have the freedom to present their concerns and that these concerns are heard and honored. … Trust builds when members of a network acknowledge the legitimacy of each other’s goals even if they differ from one’s own [4].

Proposed structures for equitable partnerships in global health research provide insight into partnerships designed to be mutually beneficial on a number of levels. The elements include co-creation, understanding and compensating for inherent inequalities to enable all partners to fully participate and benefit from interactions; communication, mutual understanding and respect for cultural norms, including religious, cultural and societal boundaries; commitment, patience, building trust and long-term commitment; and continuous review process of review and consultation to develop and refine partnership research model [5].

The above noted principles and frameworks for international partnerships are consistent with the founding goals of the Peace Corps that have been the basis of partnerships since the organization’s inception. The three goals were written into the Peace Corps’ original legislation, signed by President Kennedy in September 1961. These intertwining and equal goals are

1. to help the people of interested countries in meeting their need for trained men and women;
2. to help promote a better understanding of Americans on the part of the peoples served; and
3. to help promote a better understanding of other peoples on the part of Americans (https://www.peacecorps.gov/about/).

## Peace Corps Sustainable Partnership Model: Three Intertwining Layers

To carry out these goals, Peace Corps built a sustainable partnership model of three intertwining layers. These layers enabled the Covid-19 emergency evacuation success worldwide. They include partnerships between the US (Peace Corps) and national leaders of each country of service; between Peace Corps host country staff and their ministry counter-parts, local officials, and Peace Corps Volunteers; and between Peace Corps Volunteers and communities in which they live and work.

*First, country to country partnerships*. Peace Corps has official government to government partnerships between the US and host governments, ensuring “voluntary engagement, mutually agreed objectives, distinct accountabilities and reciprocal obligations.” [1] Negotiated and signed country agreements lay out each country’s responsibilities for the support of Peace Corps Volunteers living and working in communities. These are developed jointly at the outset of the relationship by both countries and include health, safety, and security structures as well as numbers and types of Volunteer programs. These written country agreements are recorded at the Peace Corps, the United States Department of State, and host country foreign affairs departments. These agreements are revisited periodically between the two nations.

Ongoing informal discussions, as recommended by Zaman et al., ensure that each entity is meeting its interests and obligations, engendering trust, and that Volunteers are continuing to meet individual host country national interests [5]. The countries are “members of a network acknowledging the legitimacy of each other’s goals.” [4] For example, one country’s partnership agreement outlines an education program for classroom co-teaching, another country’s partnership emphasizes a co-partnering with community health officers for strengthening malaria prevention programs, and a third country’s priority is renewable indigenous power sources for electricity.

*Second, host country staff partnerships with their ministry counter-parts, local officials, and Volunteers*. Peace Corps host country staff (both US and local) are the middle layer of country partnerships. They negotiate with Ministry personnel to ensure relevant locally-implemented program development and implementation; manage Volunteer training; work with communities preparing projects for incoming Volunteers; and support Volunteers throughout their two-year tours. Host country staff work in both local and national languages when discussing Volunteer projects. Volunteers turn to, and trust, host staff for health and safety emergencies, personal issues, and project technical guidance demonstrating that these are partnerships built on mutual respect and trust.

*Third, Volunteer partnerships with local communities*. Each Volunteer and community is a mini-partnership based on the trust and respect embodied in the national agreements. Individual Volunteers’ living with host families, sharing meals, engaging in family activities, and developing language and detailed cultural knowledge establish this core element of sustainable partnerships. Volunteers’ project goals, whether in health, education, or other technical areas of service, depend on community involvement in order to build, as noted by Mohr above, “attributes of commitment, coordination, and trust.” [3] As an example, a Volunteer’s project to further a West African Ministry of Health’s campaign for improvement in infant nutrition depended on her spending time with families and being fully immersed in the local language and culture before nutrition discussions began. She then continued frequent visits over the year discussing and supporting subtle changes to improve family nutritional goals. I spent a day with the Volunteer and three of the families with whom she worked as they described their personal relationship with the Volunteer, nutritional changes they themselves had made, and results they saw in their own children.

These personal and professional relationships continue after Volunteers return to the US and share what they learned from the families and communities in which they lived and worked. I have heard host country Ambassadors to the US say at Peace Corps Embassy events, “returned Volunteers are better ambassadors to the US for my country than I am.”

## Participatory Analysis for Community Action, Logic Project Framework, and Acceptance Model for Safety and Security: Building, Managing, Measuring Volunteer Programs and Sustaining Safety/Security

Peace Corps has worked across the three-layer partnership model described above to strengthen a sustainable framework for Volunteer service. To do this, Peace Corps has developed two community integrative models and a safety and security acceptance model. The first community integrative model, Participatory Analysis for Community Action (PACA) [6], guides sustainable project development. The second community integrative model, the Logic Model Enhance Participatory Performance (LPF or Logic Project Framework) [7] guides and measures results over time. The third model, the Acceptance Model [8] for safety and security systems, reinforces trust and transparency at national, regional, and local levels of partnership participation.

*PACA*. The PACA model has been tested and revised by Peace Corps for over 20 years with full participation at each country’s national, regional, and local level. Volunteers use the PACA approach to engage in capacity building initiatives driven by community needs. The approach empowers community members to be their own decision makers, develop the skills needed to carry out those decisions, and take the lead in improving their own lives [10]. Using the PACA method, Volunteers work with community members closely to “brainstorm, design, iterate, implement, adjust, and improve” community-led projects throughout two years of service [10].

Through the use of PACA, Volunteers develop relationships through trust, integrating into the community, and bringing different community members together (co-creation). They gather information and gain key insights by observing, learning from, and engaging community members (communication). Together they make sense of observations, insights, and discoveries to generate or improve project ideas (commitment), and they test, refine, and continue to improve project ideas (continuous review).

*Logic Project Framework or LPF*. As noted earlier, partnership roles need to be clarified, boundaries drawn, added value defined, and outcomes measured. Co-created with community members at the start of a Volunteer’s service, the LPF outlines the project situation, inputs, outputs, outcomes and impact. These components are based on the local community counterpart(s) and Volunteer jointly gathering data which is uploaded quarterly, reviewed by both Volunteers and their community based local counterpart who help design the project, the in-country Peace Corps staff, and Peace Corps Headquarters in Washington, DC. The LPF enables collaborative discussions for clarifying roles, boundaries, value, and outcomes.

Core to sustainable PACA integration and LPF project development and measurement is Volunteers’ living with host families and sharing meals, family activities, language, and daily cultural knowledge. As suggested by Mohr above, important partnership characteristics include “attributes of commitment, coordination, and trust.” [3] Peace Corps’ three month in-country pre-service training in community settings, designed primarily by host country staff, prepares Volunteers, their community-based counterparts, and host families in aspects of these partnership commitments [9].

What Volunteers bring home—new skills and insights into community public health, literacy, education, the environment, public service—are learned through their interactions in communities culturally, linguistically, and traditionally different from their pre-Peace Corps experiences. Even as PACA and the Logic Framework measure impacts of the partnerships, individual stories remain strong indicators of success.

*The Acceptance Model for Safety and Security*. To ensure Volunteer safety and security, Volunteers are trained in emergency procedures during pre-service training and supported during their two years in-country. The Peace Corps’ integrated three-layer partnership approach is essential in assuring Volunteers, host families, and community counter-parts are integrated into these safety and security systems. The Peace Corps model is based on building trusting close relationships to keep Volunteers safe.

For example, Volunteers are provided a phone pre-populated with emergency information, and then tested in emergency safety and health procedures every few months. As community and national needs and assets change, Peace Corps’ emergency plans change accordingly. Plans are adapted to local environments such as Volunteer distance from the capital city, modes of transportation, and village isolation. These plans are shared and discussed (co-creation, communication) regularly with local and national host officials to ensure full partner participation. Even the local police chief and the health clinic health official are integral to Volunteers’ health and safety (continuance and continual review).

Peace Corps’ key messages for Volunteer recruitment, host-country and American staff training, and host-country program and project development embody model practices for effective international partnership and are consistent with Peace Corps’ own 60-year partnership framework. The importance of this approach was shared with me when the Acting Peace Corps Director addressed all living former Peace Corps Directors (myself included) in 2021:

The Peace Corps is an international network of Volunteers, community members, host country partners, and staff who each have a unique service journey, defined by an enduring desire to learn and grow.

The Peace Corps’ approach, grounded in mutual respect and collaboration, is a catalyst for grassroots development.

At the invitation of governments around the world, Peace Corps Volunteers work alongside community members on locally- prioritized projects that build relationships, promote knowledge exchange, and make a lasting and measurable impact [10].

## Trusted Partnership in Practice: The Evacuation

In early spring 2020, the Covid-19 pandemic’s meteoric spread across countries required global agencies and organizations to immediately evacuate staff and participants or take other health safety measures to protect citizens while reducing the disease’s further spread. The Peace Corps had 7 000 Volunteers in communities in 61 countries in March 2020, when it ordered all Volunteers to return to the United States to protect both Volunteer health and the health of citizens in countries in which they were serving. A full Peace Corps evacuation had never happened before in its 60-year history.

At midnight on March 15, 2020, as Peace Corps Director, I sent out a communication to all Volunteers and host country staff. Included in the communication were the following statements:

I have made the difficult decision to evacuate all of our Volunteers globally. As Covid-19 continues to spread and international travel becomes more challenging by the day, we are acting now to safeguard your well-being and prevent a situation where Volunteers are unable to leave their host countries.

I also want to assure you and our host country partners that these evacuations represent the temporary suspension of Volunteer activities. We are not closing posts, and we will be ready to return to normal operations when conditions permit [11].

Undergirding my communique was Mohr et al.’s notion that “commitment, coordination, and trust; communication quality and participation; and conflict resolution technique of joint problem solving” [3]. would ensure a safe evacuation and the resumption of activities in the future. My message and actions depended on complete trust of thousands who received the message globally to jointly solve critical evacuation problems in a never before managed health emergency.

When the March 15 email was sent, everyone knew what to do based on extensive practiced procedures. They followed protocols locally prepared, regularly updated, and successfully improvised based on that country’s assets at the time.

Within three hours of the initial communication, the staff teams on the ground were in touch with country national, state, and local officials. Similar communications were sent to Embassy officials. Host country staff personally explained the decision to counterparts at all levels of government. Foreign affairs officials, national and regional safety and security officials, and community leaders were critical to the support for Volunteers as they said goodbye and moved across the country to their international flights. National leaders and ministry officials had trust that leaving was related to a global health threat and that their leadership support would ensure long term partnership in the future. As stated by Pennink, “Trust is increasingly seen as important for long-term interactions of high interdependence … trust can act as a governing mechanism in situations of high risk.” [12]

To move seven thousand Volunteers from small villages, to capital cities, airports, international and domestic flights and quarantine rooms required support and collaboration from those in communities everywhere in the world. Individuals offered help—quickly organized goodbye ceremonies for students and families, seats on buses, extra packets of food, free hostel rooms for the night, rides to train stations, airlines scheduled adjustments for Volunteers, and safety support by ministry officials. The core of the evacuation’s success was thousands of willing hands given with care and trust. With country level support and care, all Volunteers returned to the US within nine days with no one getting lost, injured, or sick.

Volunteers, host country staff, and national officials knew what to do in an emergency. For example, host staff members organized hundreds of tasks to make the evacuation logistically successful and personally heart felt. They cared about Volunteers and communities with each logistical step they took, even as they coped with their own family Covid-19 emergencies. Peace Corps headquarters assured host country staff and national leaders that Peace Corps in-country offices and all staff would continue until Volunteers returned. Peace Corps continued to honor the country agreements.

The evacuation success came directly from the Peace Corps’ years of sustainable international partnerships across all regions of the world. Nearly two years later, these commitments have continued even as Volunteers have not been able to return.

During the evacuation and immediately afterward, many staff and Volunteers wrote about their experiences during the evacuation illustrating the themes of trust. Below are three short examples, one from a Volunteer, and two from Peace Corps Country Directors supervising the in-country efforts.

I firmly believe that one-to-one relationships built at a grassroots level between people who are fundamentally different is the best pathway to world peace. But I forgot how much it hurts to leave your friends.” (Rok Locksley, Volunteer in the Philippines)

The farthest part of the country is normally a 40-hour bus ride … And of course, because it’s Mongolia, there was a blizzard. … My communities said as Volunteers left, ‘We want the Volunteers back as soon as possible’. (CD in Mongolia)

We move PCVs in six different buses, with police and regional security officer escort since it is now illegal to gather more than 10 people because of Covid-19. At Kyiv airport, there’s a technical issue: They can’t issue or print boarding passes. So, airport staff write them all out by hand.” (CD in Ukraine) (*WorldView*, NPCA, 2020)

## The Future

Throughout the evacuation and the following year, the global Peace Corps community has been attentive to each country’s own emergency health management systems, how each country cares for citizens during the pandemic, and country specific economic and educational impacts from the pandemic. Peace Corps’ return to countries in 2022 will be responsive to these changes. The agency will adapt its training, procedures, interactions with host country communities to assure the new Volunteers will be prepared for the experiences and new tasks countries request as they emerge from the global pandemic, country by country, region by region. Sustainable partnerships based on Participatory Analysis for Community Action (PACA) make possible the revised training and programming requests countries are making and will make in the future to address current community and country needs.

Even without Volunteers in the field, Peace Corps’ strong international partnerships continue. All in-country Peace Corps offices have remained open and fully staffed, with staff working at home or in offices depending on country specific Covid-19 situations. Host country staff, local counterparts, and national officials facilitate daily and weekly in-country communication with national ministries and communities. These are matched with the communication to Peace Corps headquarters. This ongoing activity in the face of uncertainty depends on trust that Volunteers will return and trust that when Volunteers return, they will be ready to meet the new challenges created by Covid-19 in the health, economic, and social sectors of the countries and communities where they will work.

## Conclusion and Reflection

I have been shaped by my own Peace Corps service 55 years ago which included time with a Tunisian host family who became my immediate family and students who became my extended family. My personal experiences have been replicated thousands of times over the years by other Peace Corps Volunteers bound together as host family and a Volunteer, head master and Volunteer, or health clinic nurse and Volunteer – all sharing humility and respect for each other as they work to achieve common goals. The Volunteer’s statement earlier, “but I forgot how much it hurts to leave your friends,” reflects the depth of personal trust developed over long-term, intentional immersion experiences. The example of airport staffers writing all the tickets out by hand shows the breadth of partnership commitment, even in one small unanticipated action. It reflects years of commitment, patience, and trust. As Peace Corps continues its planned return, it will rely on the collaboration within the three-layered partnership model. This will enable Volunteers to respond to community-defined needs and interests resulting from Covid-19’s world-wide impact on health, education, economic, and culture changes.
